# Cellular senescence in the response of HR^+^ breast cancer to radiotherapy and CDK4/6 inhibitors

**DOI:** 10.1186/s12967-023-03964-4

**Published:** 2023-02-10

**Authors:** Vanessa Klapp, Aitziber Buqué, Norma Bloy, Ai Sato, Takahiro Yamazaki, Xi Kathy Zhou, Silvia C. Formenti, Lorenzo Galluzzi, Giulia Petroni

**Affiliations:** 1 Department of Radiation Oncology, Weill Cornell Medical College, New York, NY USA; 2 Healthcare Policy and Research, Weill Cornell Medical College, New York, NY USA; 3 Sandra and Edward Meyer Cancer Center, New York, NY USA; 4 Caryl and Israel Englander Institute for Precision Medicine, New York, NY USA; 5 Department of Experimental and Clinical Medicine, University of Florence, Florence, Italy

**Keywords:** β-galactosidase, INK-ATTAC mice, MCF7 cells, MDA-MB-231 cells, MPA/DMBA-driven mammary carcinogenesis, TS/A cells

## Abstract

**Background:**

Preclinical evidence from us and others demonstrates that the anticancer effects of cyclin-dependent kinase 4/6 (CDK4/6) inhibitors can be enhanced with focal radiation therapy (RT), but only when RT is delivered prior to (rather than after) CDK4/6 inhibition. Depending on tumor model, cellular senescence (an irreversible proliferative arrest that is associated with the secretion of numerous bioactive factors) has been attributed beneficial or detrimental effects on response to treatment. As both RT and CDK4/6 inhibitors elicit cellular senescence, we hypothesized that a differential accumulation of senescent cells in the tumor microenvironment could explain such an observation, i.e., the inferiority of CDK4/6 inhibition with palbociclib (P) followed by RT (P→RT) as compared to RT followed by palbociclib (RT→P).

**Methods:**

The impact of cellular senescence on the interaction between RT and P was assessed by harnessing female INK-ATTAC mice, which express a dimerizable form of caspase 8 (CASP8) under the promoter of cyclin dependent kinase inhibitor 2A (*Cdkn2a*, coding for p16^Ink4^), as host for endogenous mammary tumors induced by the subcutaneous implantation of medroxyprogesterone acetate (MPA, M) pellets combined with the subsequent oral administration of 7,12-dimethylbenz[a]anthracene (DMBA, D). This endogenous mouse model of HR^+^ mammary carcinogenesis recapitulates key immunobiological aspects of human HR^+^ breast cancer. Mice bearing M/D-driven tumors were allocated to RT, P or their combination in the optional presence of the CASP8 dimerizer AP20187, and monitored for tumor growth, progression-free survival and overall survival. In parallel, induction of senescence in vitro, in cultured human mammary hormone receptor (HR)^+^ adenocarcinoma MCF7 cells, triple negative breast carcinoma MDA-MB-231 cells and mouse HR^+^ mammary carcinoma TS/A cells treated with RT, P or their combination, was determined by colorimetric assessment of senescence-associated β-galactosidase activity after 3 or 7 days of treatment.

**Results:**

In vivo depletion of p16^Ink4^-expressing (senescent) cells ameliorated the efficacy of P→RT (but not that of RT→P) in the M/D-driven model of HR^+^ mammary carcinogenesis. Accordingly, P→RT induced higher levels of cellular senescence than R→TP in cultured human and mouse breast cancer cell lines.

**Conclusions:**

Pending validation in other experimental systems, these findings suggest that a program of cellular senescence in malignant cells may explain (at least partially) the inferiority of P→RT versus RT→P in preclinical models of HR^+^ breast cancer.

**Supplementary Information:**

The online version contains supplementary material available at 10.1186/s12967-023-03964-4.

## Background

Cyclin-dependent kinase 4/6 (CDK4/6) inhibitors are a novel class of targeted anticancer agents with cytostatic activity that have recently been introduced in the clinical practice for the treatment of advanced/metastatic hormone receptor (HR)^+^ breast cancer, as they have demonstrated pronounced therapeutic effects in the context of manageable (primarily hematological) side effects [1]. However, while CDK4/6 inhibitors significantly extend both progression-free survival (PFS) and overall survival (OS) of women with advanced/metastatic HR^+^ breast cancer, the majority of these patients eventually progress and succumb to their disease [2–4], calling for the implementation of combinatorial regimens with superior therapeutic activity (and acceptable toxicity).

Radiation therapy (RT) has attracted considerable interest as a combinatorial partner for CDK4/6 inhibitors, for numerous biological and clinical reasons [5]. First, these two modalities may synergize since they target different phases of the cell cycle. Indeed, CDK4/6 inhibitors block cell cycle progression (in cells overexpressing CDK4 or CDK6) at the G_1_-S transition [6, 7], whereas RT mediates cytostatic/cytotoxic effects that generally emerge at the G_2_-M transition [8–10]. Second, CDK4/6 inhibitors have been shown to mediate multiple immunostimulatory effects that may contribute to their clinical activity [11–15], most of which are distinct from the immunostimulatory effects mediated by RT [16–21]. Thus, CDK4/6 inhibitors and RT are expected to engage in at least some degree of therapeutic synergism. Finally, RT is widely available and its well-defined toxicity profile renders it an optimal partner for CDK4/6 inhibitors in combinatorial clinical trials with limited safety concerns [22, 23].

We and others have shown that RT and CDK4/6 inhibitors can be safely combined and mediate additive-to-synergistic therapeutic effects in a variety of tumor models, including cultured human and mouse cancer cells, as well as human tumors xenografted in immunodeficient mice and mouse tumors evolving in immunocompetent syngeneic hosts [24–31]. Importantly, some of these studies revealed the relevance of treatment schedule on therapeutic efficacy [24–26, 32]. Specifically, while delivering RT prior to the CDK4/6 inhibitor palbociclib (P) resulted in superior tumor control as compared to RT or P employed as standalone interventions in various models of HR^+^ and HR^−^ breast cancer, such a beneficial interaction was abrogated when P was administered prior to RT [24, 32].

Cellular senescence, an irreversible cell cycle arrest that is accompanied by the abundant secretion of cytokines and other bioactive factors (a process commonly referred to as senescence-associated secretory phonotype [SASP]) [33], has been attributed beneficial as well as detrimental effects on the sensitivity of various tumors to therapy, with a considerable degree of context dependency [34, 35]. Since both RT and CDK4/6 inhibitors elicit cellular senescence as part of their in vivo effects [36–41], we hypothesized that the differential efficacy of RT followed by (→) P vs P→RT could depend on the differential accumulation of senescent cells in the tumor microenvironment (TME). Here, we demonstrate that the elimination of senescent (p16^+^) cells by a genetic approach [42] ameliorates the efficacy of P→RT (without a significant effect on RT→P) in an immunocompetent model that recapitulates key immunobiological features of human HR^+^ breast cancer. Consistent with this notion, RT→P induced less cellular senescence than P→RT in cultured human and mouse breast cancer cell lines. Thus, a program of cellular senescence may negatively influence the sensitivity of HR^+^ breast cancer to RT combined with CDK4/6 inhibitors, a possibility that awaits validation in other experimental systems.

## Methods

### Reagents and cell culture

Unless otherwise specified, reagents were obtained from Millipore Sigma. Palbociclib (#HY-50767A), and AP20187 (#HY-13992) were purchased from MedChem Express. Human mammary adenocarcinoma MCF7 cells (RRID:CVCL_0031) and triple negative breast carcinoma (TNBC) MDA-MB-231 (RRID: CVCL_0062) cells, as well as mouse mammary adenocarcinoma TS/A cells (RRID:CVCL_VQ63) were kindly provided by Dr. Sandra Demaria (Weill Cornell Medicine). All cell lines were routinely maintained at 37 °C under 5% CO2, in Dulbecco's Modified Eagle's Medium (DMEM) supplemented with 10% fetal bovine serum (FBS), 5 mM L-glutamine, 5 mM HEPES buffer, 50 μM β-mercaptoethanol 100 U mL^−1^ penicillin sodium, 100 µg mL^−1^ streptomycin sulfate and 50 µg mL^−1^ gentamycin. Cells were authenticated by STR profiling (a service provided by IDEXX Bioresearch) and periodically checked for *Mycoplasma spp.* contamination by the PCR-based LookOut® Mycoplasma PCR Detection Kit. All cells were employed for experiments 2–10 passages after thawing. All irradiation procedures were performed on a Small Animal Radiation Research Platform (SARRP, from Xstrahl).

### β-galactosidase assays

Cellular senescence was assessed by colorimetric staining of senescence-associated β-galactosidase activity with the Cellular Senescence Assay (#KAA002, for MCF7 and MDA-MB-231 cells) or the Senescence β-Galactosidase Staining Kit (#9860, Cell Signaling Technology, for TS/A cells), as per the manufacturer’s instructions. Stained cells were imaged on an ECLIPSE Ti Inverted Microscope System (Nikon) controlled by NIS-Elements AR v. 4.11.00 (Nikon) at 10X magnification, 4 brightfield images per well. Images were manually counted for staining positivity on ImageJ2 (RRID:SCR_003070) v. 2.3.0 (NIH).

### In vivo* experiments*

INK-ATTAC mice, which express a dimerizable variant of caspase 8 (CASP8) under the promoter of cyclin dependent kinase inhibitor 2A (*Cdkn2a,* coding for p16^Ink4^) [42], were a kind gift from Jan Van Deursen (Unity Biotechnology). All mice were maintained in specific pathogen-free conditions, and all experiments were performed according to the common Guidelines for the Care and Use of Laboratory Animals. Specifically, all animal experiments were approved by the Institutional Animal Care and Use Committee (IACUC) of Weill Cornell Medical College (#2017-0012, #2019-0022). Endogenous mammary tumors were established as per conventional procedures [43]. In brief, 6–9 weeks old female were surgically implanted *s.c.* with 50 mg slow-release (90 days) medroxyprogesterone acetate (MPA, M) pellets (Innovative Research of America, #NP-161) followed by oral administration of 1 mg 7,12-dimethylbenz[a]anthracene (DMBA, D) in 200 µL corn oil once a week on weeks 1, 2, 3, 5, 6 and 7 after implantation of the MPA pellet (week 0) [43]. Next, mice were routinely assessed to detect palpable tumors along the milk lines, which were allowed to reach a surface area of 15–45 mm^2^ (d0). On d0, mice were randomly allocated to receive (1) no treatment; (2) focal RT (3 fractions of 10 Gy each on d0–d2), (3) oral palbociclib (100 mg/Kg in 50 mM sodium lactate pH 4.0, on d0–d13), (4) focal RT (on d0-d2) followed by oral palbociclib (on d3–d16); (5) oral palbociclib (on d0–d13) followed by focal RT (on d14–d16), optionally in the context of intraperitoneal 2 mg/Kg AP20187 (starting on d0, then every 3 days until endpoint). Mice were monitored for signs of toxicity (weight loss, anorexia, hunched posture), growth of the primary (target) lesion (by a common caliper) as well as emergence and growth of secondary tumors. Tumor surface was calculated as the area of an ellipse (A = longest diameter X shortest diameter X π/4), as per common procedures [43]. Mice were euthanized when lesions reached 180–200 mm^2^ cumulative surface area, which was employed as surrogate marker for overall survival (OS). Progression-free survival (PFS) was defined as the time from treatment initiation to progression of the primary tumor (relative surface area > 1.440) or the appearance of secondary lesions with surface area > 20 mm^2^. Additional parameters that were investigated include surface area of primary and secondary tumors from the day of detection and impact of secondary tumors on disease burden at endpoint.

### Statistical analysis

Unless otherwise specified, data management, analysis and graphing were performed with Prism v. 8.4 (GraphPad, RRID:SCR_002798) or Excel 365 ProPlus (Microsoft, RRID:SCR_016137). Figures were prepared with Illustrator 2022 (Adobe, RRID:SCR_010279). Unless otherwise indicated, in vitro results were obtained from at least three independent biological samples collected over at least two independent experiments. A linear mixed effects model with treatment group, time, treatment group by time interaction as the fixed effects and random intercept and slope by mouse was applied to model tumor growth using R version 4.2.0 (R Core Team, RRID:SCR_001905) and the R packages nlme [44] and multcomp [45]. Tumor areas were square root-transformed to ensure underlying model assumptions were satisfied. Heterogeneity in variances were modeled with applying time-dependent weights. Tumor growth rates were compared for contrasts of interest using simultaneous tests for general linear hypotheses. *p* values were not adjusted for multiple comparisons for the analysis of tumor growth. Statistical significance on PFS and OS was assessed by the Gehan-Breslow-Wilcoxon test. Statistical significance on relative impact of secondary tumor burden at endpoint were assessed by Kruskal–Wallis plus uncorrected Dunn's test. Linear mixed-effects regression was used to estimate percent β-gal^+^ cells in each treatment group while accounting for potential within experiment and within technical replicate correlations. Heteroscedasticity in within-group errors was modeled by allowing different variances for different groups through group specific weights. Simultaneous tests for generalized linear hypotheses was used to evaluate contrasts of interest. *p* values were not adjusted for multiple comparisons for the analysis of β-gal^+^ cells.

## Results

### Elimination of senescent cells enables therapeutic synergy by P→RT

**Fig. 1:**
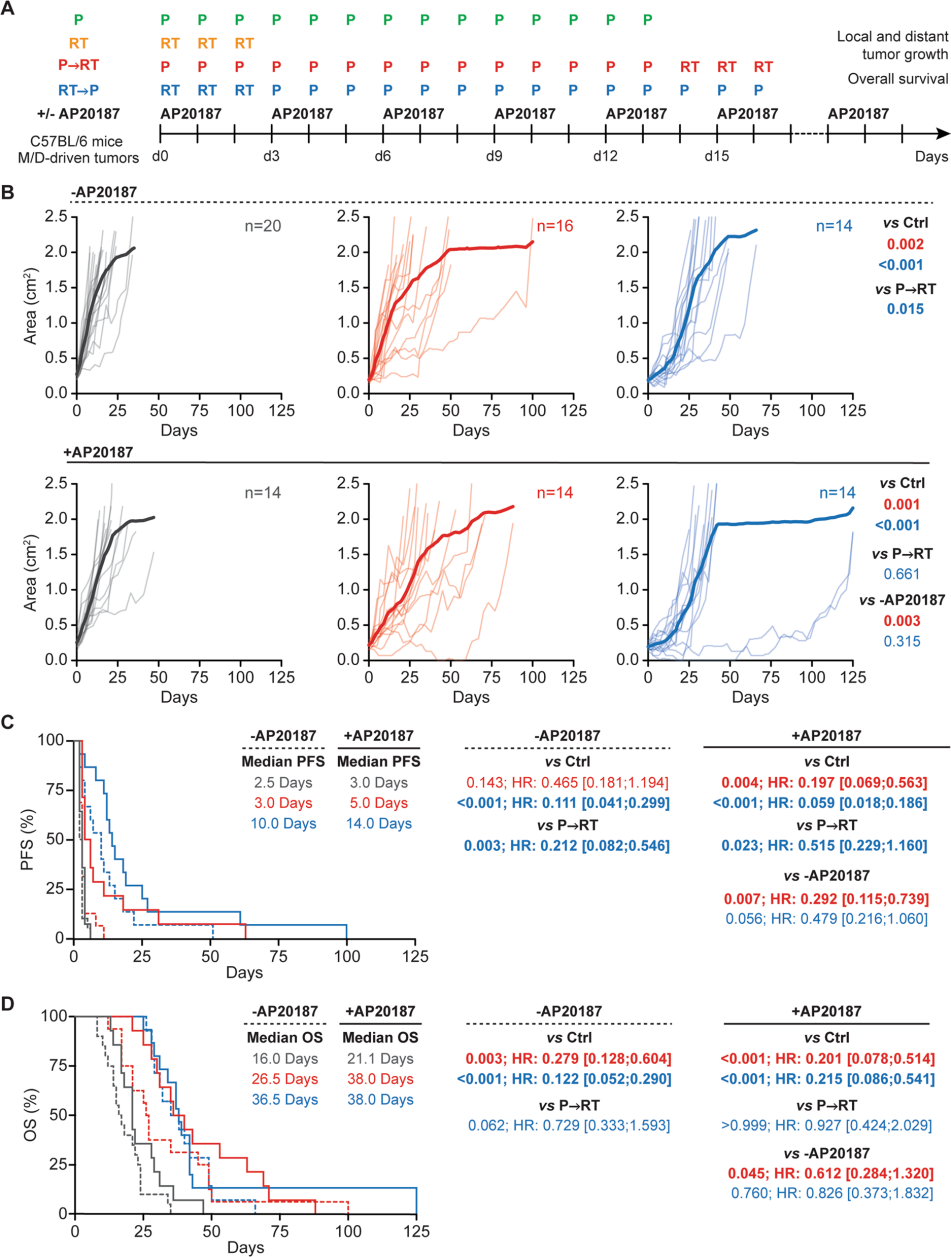
Elimination of senescent cells restores superior therapeutic effects by P→RT. Immunocompetent female INK-ATTAC mice bearing palpable M/D-driven tumors were randomly to allocated (1) no treatment; (2) focal radiation therapy (RT), (3) palbociclib (P) and their combination, optionally in the context of AP20187 administration, as indicated (**a**). Mice were followed for tumor growth and euthanatized when cumulative tumor surface reached 180–200 mm^2^, which was used to define overall survival (OS). Individual growth curves for cumulative tumors (**b**), progression-free survival (PFS, **c**) and OS (**d**) are reported. Differences in tumor growth (**b**) were assessed for statistical significance by a linear mixed effects model followed by simultaneous tests of general linear hypotheses Differences in PFS (**c**) and OS (**d**) were assessed for statistical significance by Gehan-Breslow-Wilcoxon test. Number of mice, hazard ratio (HR) with 95% confidence interval and *p* values are reported

To assess the impact of cellular senescence on the interaction between RT and P, we harnessed female INK-ATTAC mice, which express a dimerizable form of caspase 8 (CASP8) under the promoter of cyclin dependent kinase inhibitor 2A (*Cdkn2a,* coding for p16^Ink4^) [42], as host for endogenous mammary tumors driven by medroxyprogesterone acetate (MPA, M) pellets plus 7,12-dimethylbenz[a]anthracene (DMBA, D) [43] that were allocated to RT, P or their combination in the optional presence of the CASP8 dimerizer AP20187 (Fig. 1A). In immunocompetent female C57BL/6 mice, M/D-driven tumors recapitulate multiple immunobiological features of HR^+^HER2^−^ breast cancer in women, including a scarce immune infiltrate [43], pronounced resistance to immune checkpoint inhibition with programmed cell death 1 (PDCD1, best known as PD-1) blockers [43], and exquisite sensitivity to CDK4/6 inhibitors [24], representing a unique model for translational studies.

Confirming our previous findings [24], both focal RT administered in 3 daily fractions of 10 Gy each (non-ablative) and oral P (100 mg/Kg daily, for 14 days) mediated single-agent therapeutic efficacy against M/D-driven carcinomas, manifesting with cumulative tumor growth delay (Additional file 1: Fig. S1A,B), increased PFS (defined by the progression of the primary tumor—relative surface area > 1.440—or the appearance of secondary lesions with surface area > 20 mm^2^) (Additional file 1: Fig. S1C) and OS extension from a median of 16.0 days (untreated tumors) to a median of 37.5 days (RT) or 29.0 days (P) (Additional file 1: Fig. S1D). As previously shown [24], the RT→P regimen was superior to the P→RT regimen at delaying tumor growth (Fig. 1B) and extending PFS (Fig. 1C), although this was associated with only a trend toward improved OS benefit (Fig. 1D). Most, likely, this was due to the emergence of secondary malignancies (which are common during M/D-driven carcinogenesis) [43] that were never irradiated and not necessarily exposed to P (depending on time of detection) but contributed to systemic tumor burden (the determinant of OS in this setting) (Additional file 2: Fig. S2A,B). AP20187 administration had virtually no effects on M/D-driven carcinomas receiving RT or P as standalone interventions (Additional file 1: Fig. S1A-D, Additional file 1: Fig. S2C), perhaps with the exception of a slight delay in secondary tumor growth upon irradiation (Additional file 2: Fig. S2D). Similarly, AP20187 failed to affect tumor growth, PFS and OS in mice with M/D-driven tumors receiving RT→P (Fig. 1B–D). Conversely, elimination of senescent cells with AP20187 improved the ability of P→RT to mediate systemic disease control and increase both PFS and OS (Fig. 1B–D).

Taken together, these findings suggest that a program of cellular senescence may contribute to the reduced efficacy of P→RT over RT→P in controlling M/D-driven carcinomas developing in immunocompetent mice.

## Treatment schedule affects induction of senescence by RT plus P

**Fig. 2:**
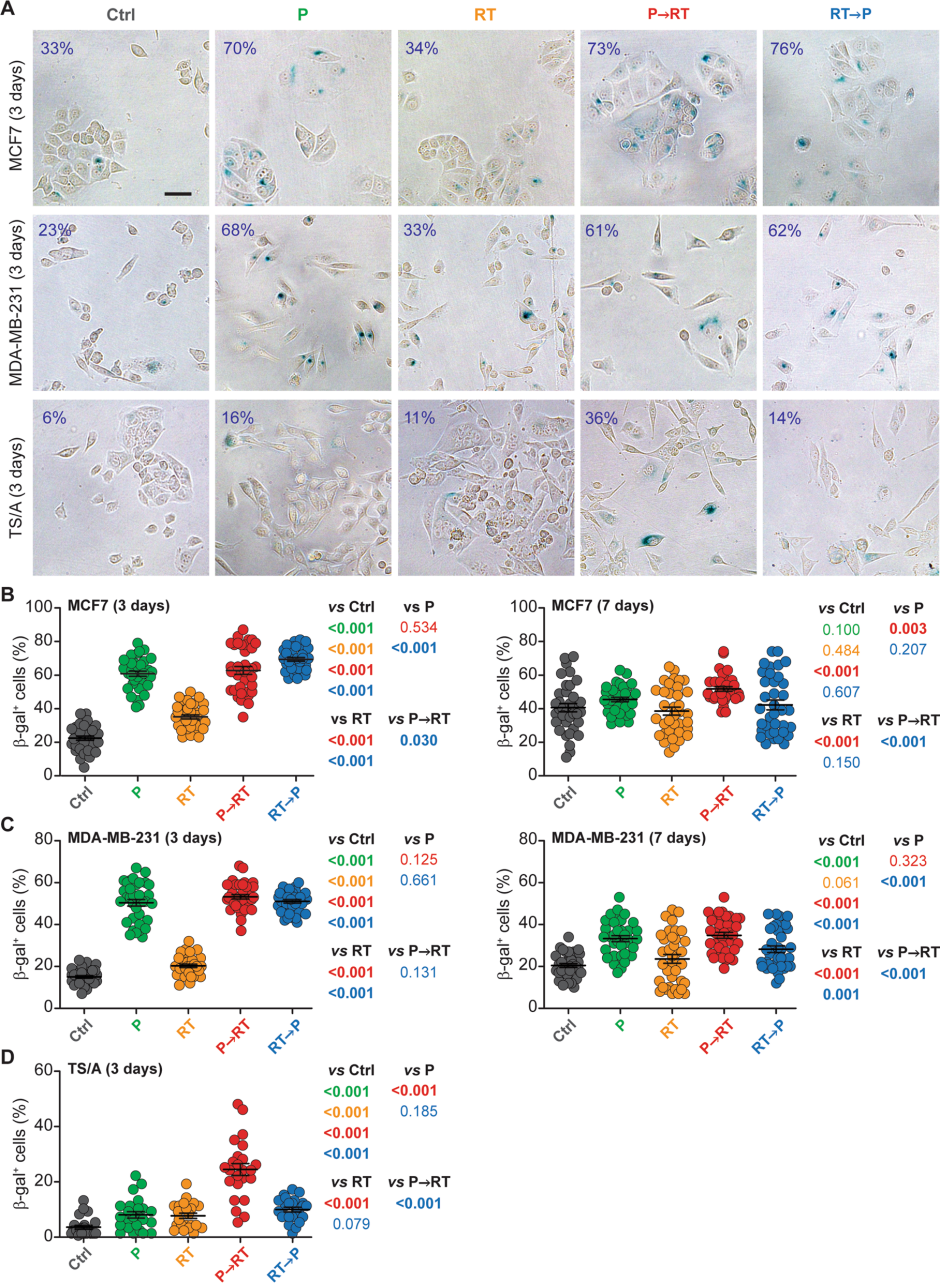
Treatment schedule affects induction of senescence by RT plus P. Human MCF7, MDA-MB-231 and mouse TS/A cells were cultured in control conditions or exposed to radiation therapy (RT), palbociclib (P) or their combination, followed by the assessment of cellular senescence upon colorimetric senescence-associated β-galactosidase (β-gal) assessment. Representative images taken 3 days from treatment initiation (**a**) and quantitative data (mean ± SEM plus individual data points) obtained 3 or 7 days after treatment initiation for MCF7 (**b**), MDA-MB-231 (**c**), and TS/A cells (**d**) are reported. Results are from 2–3 independent experiments each encompassing 3 technical replicates and 4 images per condition. Differences were evaluated for statistical significance by linear mixed-effects regression followed by simultaneous tests of general linear hypotheses; *p* values are reported. Percentage of β-gal^+^ cells is indicated in the upper left corner of each representative image. Scale bar: 50 µm

To investigate the impact of treatment schedule on the induction of cellular senescence, we exposed cultured human mammary HR^+^ adenocarcinoma MCF7 cells and triple negative breast carcinoma (TNBC) MDA-MB-231 cells to RT (a single fraction of 1 Gy), 100 nM P or their combination (as per previously reported combinatorial schedules) [24], followed by the colorimetric assessment of senescence-associated β-galactosidase (β-gal) activity 3 and 7 days later. At the 3 days endpoint, both RT and P induced a statistically significant increase in the percentage of β-gal^+^ MCF7 and MDA-MB-231 cells as compared to control conditions, although the magnitude of this effect was much more pronounced for P (Fig. 2A–C). Accordingly, adding RT to P failed to increase the percentage of β-gal^+^ MCF7 and MDA-MB-231 cells over P alone, irrespective of treatment schedule (Fig. 2A–C), with the exception of a minor but statistically significant increase in MCF7 cells treated with RTP vs P alone (Fig. 2B). At the 7 days endpoint, the ability of RT and P to elicit senescence-associated β-galactosidase over control conditions was completely lost in MCF7 cells (Fig. 2B), potentially reflecting an increase in senescence-associated β-galactosidase positivity at baseline (Fig. 2B). Conversely, P (but not RT) employed as a standalone agent was associated with an increase in β-gal^+^ MDA-MB-231 cells also at the 7 days endpoint (Fig. 2C). Importantly, in the MCF7 model, only P→RT (but not RT→P) caused an accumulation of β-gal^+^ cells above control levels 7 days after treatment (Fig. 2B). Moreover, in the MDA-MB-231 model, both combinatorial regimens elicited senescence-associated β-gal activity 7 days after treatment, but this effect was considerably more pronounced for P→RT over RT→P (Fig. 2C). Similar effects were documented in mouse HR^+^ mammary carcinoma TS/A cells exposed to 500 nM P, a single RT fraction of 4 Gy or their combinations and assessed for β-gal positivity 3 days later (Fig. 2A, D).

These data suggest that the induction of cellular senescence by RT and P combinations is sensitive to administration schedule.

## Conclusions

Here, we demonstrate that removing p16^+^ senescent cells has a positive impact on the efficacy of hypofractionated RT combined with P in a highly translational model of HR^+^ breast cancer, but only when RT and P are combined according to the P→RT schedule (Fig. 1). Consistently, P→RT was found to induce increased levels of cellular senescence in cultured human and mouse mammary adenocarcinoma cells as compared to RT→P (Fig. 2). Taken together, these data suggest that a differential accumulation of senescent cells in the TME may contribute to the inferiority of P→RT over R→TP in preclinical models of HR^+^ BC [24]. Similar observations have previously been made in mouse models of TNBC treated with chemotherapy [46]. However, senescence as induced by CDK4/6 inhibitors (alone or combined with other agents including MEK inhibitors) has consistently been associated with beneficial and/or therapeutically actionable immunological alterations of the TME [47–49]. Moreover, specific programs of cellular senescence have been recently linked to improved activation of tumor-targeting immune responses downstream of superior antigen presentation [50]. Thus, the P→RT approach may result not only in increased levels of senescence, but also in qualitative alterations of the SASP that may negatively affect therapeutic responses. At least in part, this may reflect the ability of M/D-driven carcinomas to evade natural killer (NK) cell-dependent immunosurveillance [43], knowing that NK cells appear to be particularly active at eliminating senescent (pre-)malignant cells [51, 52].

Further supporting this possibility, RT, P and P→RT had comparable activity on tumor growth in our model (Fig. 1), yet elimination of p16^+^ senescent cells was beneficial only in the latter therapeutic scenario. Moreover, quantitative differences in senescence induction by RT, P and their combination in vitro were limited (Fig. 2). Non-malignant p16^+^ components of the TME may also contribute to the inferiority of P→RT over RT→P in our model. For instance, RT is well known to cause stromal fibrosis upon the induction of cellular senescence [53, 54], and senescence in the stroma establishes a robustly immunosuppressive microenvironment that (at least in some models) promotes and sustains tumor growth [54, 55].

Unfortunately, whether similar effects may occur in patients with HR^+^HER2^−^ breast cancer can only be partially investigated. Indeed, while some clinical trials combining RT and CDK4/6 inhibitors are open, including studies that involve the collection of biopsies after relapse such as the CIMER trial (NCT04563507), none of them aims at comparing distinct treatment schedules, with a majority of ongoing studies adopting a concurrent or RT-first approach (source www.clinicaltrials.gov). Despite this and other unknowns, our data suggest that a program of cellular senescence may influence the response of HR^+^HER2^−^ breast cancer to RT when combined with CDK4/6 inhibitors. Such a possibility warrants independent validation in other experimental systems.

## Supplementary Information

**Additional file 1: Figure S1.** Elimination of senescent cells does not affect the therapeutic effect of palbociclib and RT. Immunocompetent female INK-ATTAC mice bearing palpable M/D-driven tumors were randomly to allocated (1) no treatment; (2) focal radiation therapy (RT), (3) palbociclib (P), optionally in the context of AP20187 administration, as indicated (a). Mice were followed for local and distant tumor growth and euthanatized when cumulative tumor surface reached 180-200 mm2, which was used to define overall survival (OS). Individual growth curves for cumulative disease burden (b), progression-free survival (PFS, c) and OS (d) are reported. Differences in tumor growth (b) were assessed for statistical significance by a linear mixed effects model followed by simultaneous tests of general linear hypotheses. Differences in PFS (c) and OS (d) were assessed for statistical significance by Gehan-Breslow-Wilcoxon test. Number of mice, hazard ratio (HR) with 95% confidence interval and *p* values are reported.

**Additional file 2: Figure S2.** Impact of primary and secondary M/D-driven tumors on disease burden. Immunocompetent female INK-ATTAC mice bearing palpable M/D-driven tumors were randomly to allocated (1) no treatment; (2) focal radiation therapy (RT), (3) palbociclib (P), optionally in the context of AP20187 administration, as indicated (a). Mice were followed for local and distant tumor growth and euthanatized when cumulative tumor surface reached 180-200 mm2. Relative impact of secondary tumor burden at endpoint (b), as well as individual growth curves for primary (c) and secondary (d) disease burden are reported. Differences in relative impact of secondary tumor burden at endpoint (b) were assessed by Kruskal-Wallis + uncorrected Dunn's test. Differences in tumor growth (c,d) were assessed for statistical significance by a linear mixed effects model followed by simultaneous tests of general linear hypotheses. Number of mice and *p* values are reported.

## Data Availability

Not applicable.

## Abbreviations

β-gal: β-Galactosidase
CDK4/6: Cyclin-dependent kinase 4/6
D: 7,12-Dimethylbenz[a]anthracene
HR: Hormone receptor
M: Medroxyprogesterone acetate
OS: Overall survival
P: Palbociclib
PFS: Progression-free survival
RT: Radiation therapy
TME: Tumor microenvironment
