# Supplementary figures and images for: Cellular senescence in the response of HR+ breast cancer to radiotherapy and CDK4/6 inhibitors
Source: J Transl Med. 2023 Feb 10;21:110. doi: 10.1186/s12967-023-03964-4 (PMC9921325; doi:10.1186/s12967-023-03964-4)

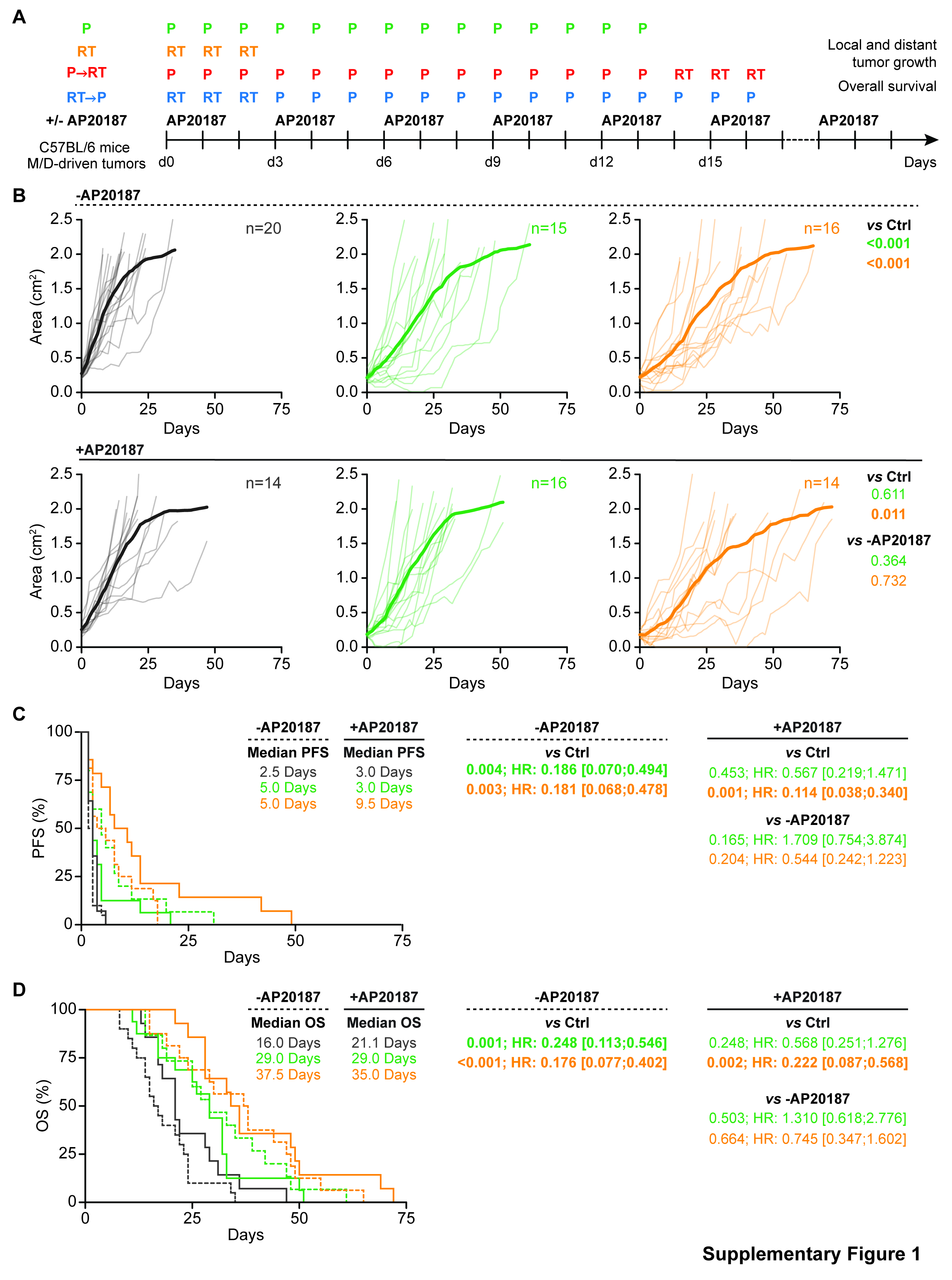

Supplement: Supplementary file 1 — Additional file 1: Figure S1. Elimination of senescent cells does not affect the therapeutic effect of palbociclib and RT. Immunocompetent female INK-ATTAC mice bearing palpable M/D-driven tumors were randomly to allocated (1) no treatment; (2) focal radiation therapy (RT), (3) palbociclib (P), optionally in the context of AP20187 administration, as indicated (a). Mice were followed for local and distant tumor growth and euthanatized when cumulative tumor surface reached 180-200 mm2, which was used to define overall survival (OS). Individual growth curves for cumulative disease burden (b), progression-free survival (PFS, c) and OS (d) are reported. Differences in tumor growth (b) were assessed for statistical significance by a linear mixed effects model followed by simultaneous tests of general linear hypotheses. Differences in PFS (c) and OS (d) were assessed for statistical significance by Gehan-Breslow-Wilcoxon test. Number of mice, hazard ratio (HR) with 95% confidence interval and p values are reported. [file 12967_2023_3964_MOESM1_ESM.tif]

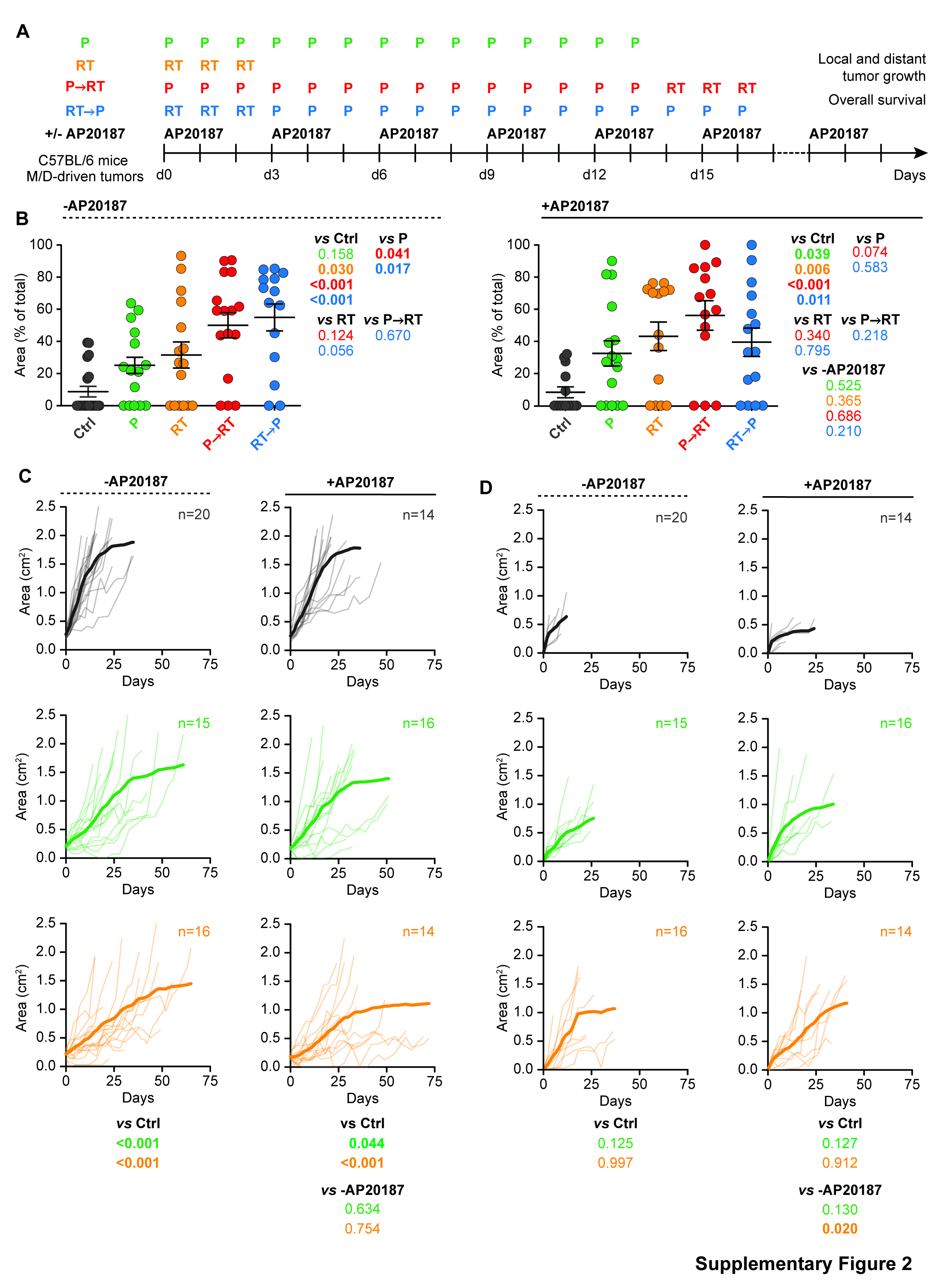

Supplement: Supplementary file 2 — Additional file 2: Figure S2. Impact of primary and secondary M/D-driven tumors on disease burden. Immunocompetent female INK-ATTAC mice bearing palpable M/D-driven tumors were randomly to allocated (1) no treatment; (2) focal radiation therapy (RT), (3) palbociclib (P), optionally in the context of AP20187 administration, as indicated (a). Mice were followed for local and distant tumor growth and euthanatized when cumulative tumor surface reached 180-200 mm2. Relative impact of secondary tumor burden at endpoint (b), as well as individual growth curves for primary (c) and secondary (d) disease burden are reported. Differences in relative impact of secondary tumor burden at endpoint (b) were assessed by Kruskal-Wallis + uncorrected Dunn's test. Differences in tumor growth (c,d) were assessed for statistical significance by a linear mixed effects model followed by simultaneous tests of general linear hypotheses. Number of mice and p values are reported. [file 12967_2023_3964_MOESM2_ESM.tif]
